# Estimation of Respiratory Rate from Functional Near-Infrared Spectroscopy (fNIRS): A New Perspective on Respiratory Interference

**DOI:** 10.3390/bios12121170

**Published:** 2022-12-14

**Authors:** Naser Hakimi, Mohammad Shahbakhti, Sofia Sappia, Jörn M. Horschig, Mathijs Bronkhorst, Marianne Floor-Westerdijk, Gaetano Valenza, Jeroen Dudink, Willy N. J. M. Colier

**Affiliations:** 1 Artinis Medical Systems, B.V., Einsteinweg 17, 6662 PW Elst, The Netherlands; 2 Department of Neonatology, Wilhelmina Children’s Hospital, University Medical Center Utrecht, Lundlaan 6, 3584 EA Utrecht, The Netherlands; 3 Biomedical Engineering Institute, Kaunas University of Technology, K. Barsausko 59, LT-51423 Kaunas, Lithuania; 4 Bioengineering and Robotics Research Center E. Piaggio and the Department of Information Engineering, School of Engineering, University of Pisa, Via G. Caruso 16, 56122 Pisa, Italy

**Keywords:** fNIRS, respiratory rate, estimation, signal quality index, physiological interference

## Abstract

Objective: Respiration is recognized as a systematic physiological interference in functional near-infrared spectroscopy (fNIRS). However, it remains unanswered as to whether it is possible to estimate the respiratory rate (RR) from such interference. Undoubtedly, RR estimation from fNIRS can provide complementary information that can be used alongside the cerebral activity analysis, e.g., sport studies. Thus, the objective of this paper is to propose a method for RR estimation from fNIRS. Our primary presumption is that changes in the baseline wander of oxygenated hemoglobin concentration ($O_{2}Hb$) signal are related to RR. Methods: fNIRS and respiratory signals were concurrently collected from subjects during controlled breathing tasks at a constant rate from 0.1 Hz to 0.4 Hz. Firstly, the signal quality index algorithm is employed to select the best $O_{2}Hb$ signal, and then a band-pass filter with cut-off frequencies from 0.05 to 2 Hz is used to remove very low- and high-frequency artifacts. Secondly, troughs of the filtered $O_{2}Hb$ signal are localized for synthesizing the baseline wander (S1) using cubic spline interpolation. Finally, the fast Fourier transform of the S1 signal is computed, and its dominant frequency is considered as RR. In this paper, two different datasets were employed, where the first one was used for the parameter adjustment of the proposed method, and the second one was solely used for testing. Results: The low mean absolute error between the reference and estimated RRs for the first and second datasets (2.6 and 1.3 breaths per minute, respectively) indicates the feasibility of the proposed method for RR estimation from fNIRS. Significance: This paper provides a novel view on the respiration interference as a source of complementary information in fNIRS.

## 1. Introduction

Over the last 20 years, functional near-infrared spectroscopy (fNIRS) has arisen as an effective optical neuroimaging modality for measuring oxygenated ($O_{2}Hb$) and deoxygenated (HHb) hemoglobin concentrations, associated with the neuronal activity [1,2]. Compared to other neuroimaging techniques such as electroencephalography (EEG) and functional magnetic resonance imaging (fMRI), fNIRS provides better spatial and temporal resolutions, respectively [3,4]. Thus, a wide range of studies in different cognitive tasks and clinical settings have employed fNIRS, e.g., [5,6,7,8].

Beside the EEG, due to its portable and non-invasive nature, fNIRS has been also used for outdoor applications [9]. In particular, the emergence of lightweight low-channel fNIRS equipment has provided a new possibility for non-laborious investigations, e.g., sport studies [10,11,12,13]. Nevertheless, the susceptibility of fNIRS to artifacts that are stemmed from various sources causes a great challenge for the accurate analysis of brain activity [14].

Generally, artifacts in fNIRS are classified into two categories: external and physiological interference [15,16]. The most prominent example of the former is motion artifacts, manifested by abrupt changes in the signal [17,18]. The latter is the interference originated from physiological systemic activities such as heart rate, blood pressure, Mayer waves, and respiration [19].

Nevertheless, despite a majority of studies that have considered physiological interference as source of artifacts in fNIRS, a few investigations have showed that such an interference can provide useful information for enhancing the accuracy of cerebral activity analysis. For example, Svinkunaite et al. [20] showed that using cardiac and respiratory features extracted from the fNIRS spectrum can enhance the accuracy of mental workload classification when employed alongside fNIRS temporal analysis. More interestingly, Hakimi et al. [21] showed the synergy of combining fNIRS temporal analysis alongside the extracted heart rate variability (HRV) for the stress assessment. According to the reported results by the authors, employing the extracted HRV from fNIRS improved the accuracy of classification by 10%.

Besides the heart rate, respiration is another physiological interference that is vividly observable in the fNIRS spectrum (usually ranging from 0.2 to 0.4 Hz) [22]. According to the best of our knowledge, no research has yet considered the possibility of respiratory rate (RR) estimation from fNIRS. This is while fNIRS is being employed in several applications where RR can also play an important role, e.g., meditation [23], stress assessment [24], and exercise [10]. Unarguably, such an estimation can provide complementary information to be used in conjunction with the cerebral activity analysis.

Motivated accordingly, we propose a new method for estimating RR from fNIRS. Inspired by the studies that estimated RR from the photoplethysmography (PPG) [25], our hypothesis is that the baseline wander of fNIRS also might be related to alternations in the respiration. On the other hand, it has been shown that respiration has a stronger influence on the $O_{2}Hb$ than the HHb signal [26]. Hence, the basis of our method is to (i) extract the troughs of the $O_{2}Hb$ signal, (ii) synthesize the baseline wander using the cubic spline interpolation of the extracted troughs, and (iii) find the dominant frequency of the baseline wander to estimate RR. In order to assess the performance of the proposed method, two different datasets are used. The first one is used to adjust the parameters of proposed method whereas the second one is only used for testing.

## 2. Methods

**Figure 1:**
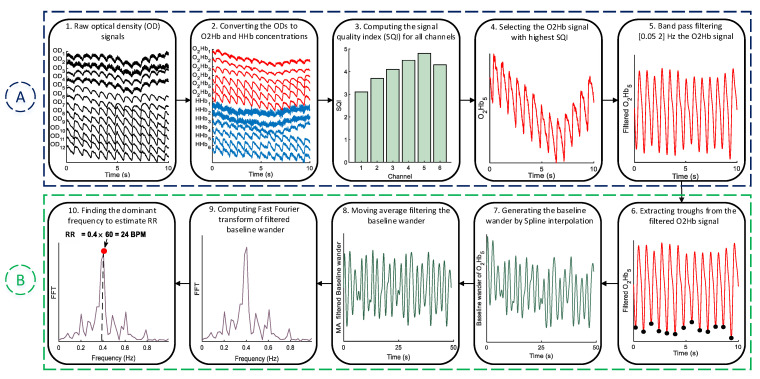
The block diagram of the proposed method. It should be noted that for the sake of clarity, fNIRS signals are shown only for 10 s.

The block diagram of the proposed method for RR estimation from fNIRS signals is shown in Figure 1. It mainly consists of two stages: pre-processing (A) and RR estimation (B). In the subsections below, each step of the proposed method is explained in detail.

### 2.1. Pre-Processing

One the most important steps in every fNIRS-based study is to find high quality data for the analysis [27]. Thus, we have employed the signal quality index (SQI) [28] algorithm, which quantitatively scores fNIRS data in a numeric scale from 1 (very low quality) to 5 (very high quality). To compute the SQI, firstly, the modified Beer-Lambert law [29] is applied to covert the optical density (OD) signals into $O_{2}Hb$ and HHb changes in concentration. Secondly, OD, $O_{2}Hb$, and HHb signals are detrended by subtracting the least-squares fit of a straight line to the data. Thirdly, a 208th-order zero-phase FIR band-pass filter with cutoff frequencies at 0.4 Hz and 3 Hz is applied on the signals from the previous step. Finally, several features are extracted from the filtered signals, and each channel is scored numerically between 1 and 5. It should be also noted that the SQI is computed based on 10 s windows. For a more detailed explanation, see [28]. After finding the highest quality channel, a zero-phase FIR band-pass filter with cut-off frequencies between 0.05 and 2 Hz is used for the removal of very low- and high-frequency artifacts from the selected $O_{2}Hb$ signal via the SQI algorithm.

### 2.2. RR Estimation

#### 2.2.1. Trough Detection

**Figure 2:**
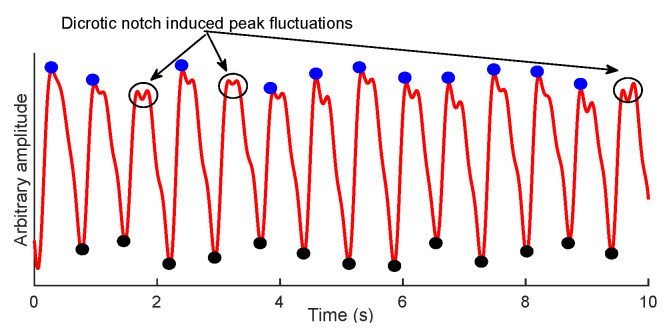
An example of $O_{2}Hb$ signal with its corresponding peaks (blue) and troughs (black).

Inspired from the PPG-based studies to estimate RR [25,30], our assumption is that alternations in the baseline wander of $O_{2}Hb$ signal can be related to RR. To this end, the fiducial points of $O_{2}Hb$ signal, i.e., the peaks and troughs, can be used. As shown in Figure 2, the synthesis of the baseline wander from troughs, compared to peaks, are more convenient as they are not subjected to dicrotic notch-induced peak fluctuations.

**Figure 3:**
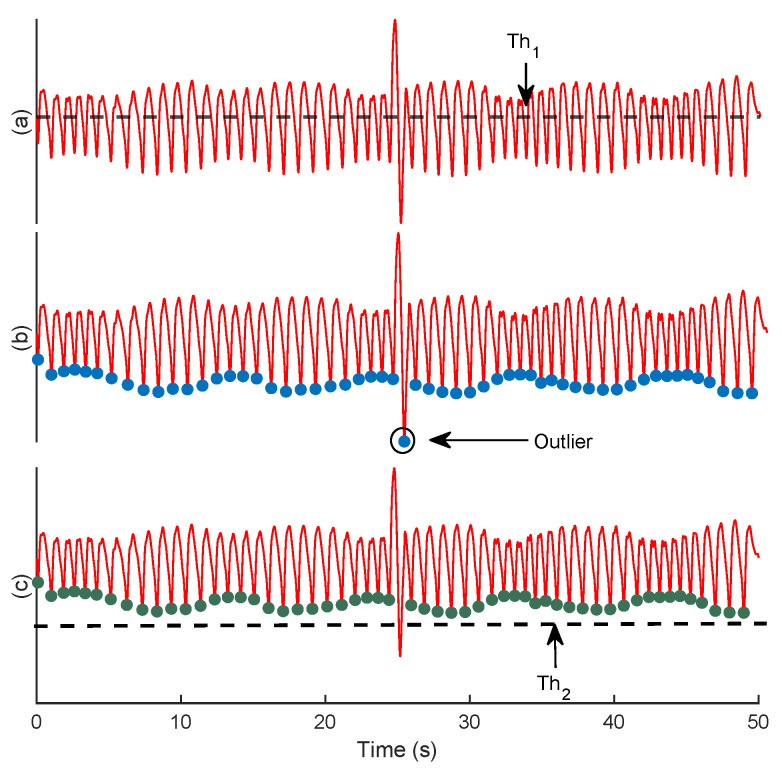
An example of the trough detection. The filtered $O_{2}Hb$ signal (**a**), the selected troughs after employing $Th_{1}$ (**b**), and $Th_{2}$ (**c**).

To localize the troughs, firstly, the filtered $O_{2}Hb$ signal, x(n), is normalized to between −1 and 1. Then, the local minima that have a value lower than $Th_{1}=A\times \mathrm{Mean}\;\left(x\left(n\right)\right)$ are considered as the potential troughs (Figure 3a). Yet, the emergence of motion artifacts can still jeopardize the accurate synthesis of baseline wander (Figure 3b). To overcome this problem, after localizing the troughs, their corresponding magnitudes are set on a vector, z(n), and elements with values lower than $Th_{2}=\mathrm{Mean}\;\left(z\left(n\right)\right)+B\times \mathrm{std}\;\left(z\left(n\right)\right)$ are discarded (Figure 3c). The coefficients *A* and *B* are constants that will be regulated empirically (see Section 4.1.1). The main steps of trough detection are summarized in Algorithm 1.
**Algorithm 1** Localization of $O_{2}Hb$ signal troughs**Input:** $O_{2}Hb$ signal x(n), constants *A*, *B*
**Output:** Troughs, *K**Initialisation* Th1← 0, Th2← 0, J← [ ], K← [ ]
1:x(n)← Normalize (x(n))
2:Th1← A × Mean (x(n))
3:**for** i=2 to length(x(n))−1 **do**
4:   **if** x(i)<x(i−1)&&x(i)<x(i+1)&&x(i)<Th1 **then**
5:     J←[Ji]
6:   **end if**
7:**end for**8:z(n)←x(J)9:Th2← Mean (z(n)) + B × std (z(n))
10:**for** i=1 to length(z(n)) **do**
11:   **if** z(i)>Th2 **then**
12:     K=[Ki]
13:   **end if**
14:**end for**15:**return***K*

#### 2.2.2. Forming the Baseline Wander Signal

After extracting the troughs, the corresponding time series, i.e., baseline wander, needs to be re-sampled. This is a necessary step, as the baseline wander generated from the troughs is irregularly sampled, whereas the following analysis needs a regularly sampled signal [25]. For this aim, the cubic spline interpolation method is employed, which approximates a signal by connecting a series of points through a polynomial equation that passes through all of those points continuously.

#### 2.2.3. FFT for RR Estimation

**Figure 4:**
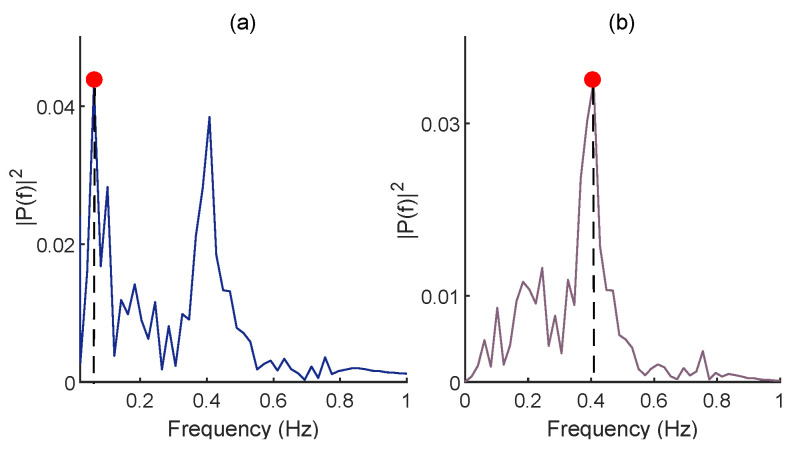
The FFT of the baseline wander before (**a**) and after (**b**) employing the MA filtering. The red dot stands for dominant frequency in the FFT domain. Note that the reference RR is 0.4 Hz in this example.

After synthesizing the baseline wander, non-respiratory oscillations, i.e., very-low-frequency components, should be filtered, as they can hinder the identification of the dominant frequency in the FFT domain (Figure 4a) [25,31]. For this aim, a moving average (MA) filter is used. After MA filtering, the dominant frequency of baseline wander (Figure 4b) is multiplied by 60 to estimate RR in breaths per minute (BPM). Although it can be argued that applying the MA may also influence the baseline wander, the MA filtering removes very-low-frequency components (below 0.04 Hz) that are not in the frequency range of RR for healthy subjects. The regulation of the moving average filter’s length is described in Section 4.1.2. The summary of RR estimation procedure after extracting the troughs of $O_{2}Hb$ signal is presented in Algorithm 2.
**Algorithm 2** Estimation of the RR from the $O_{2}Hb$ signal’s baseline wander**Input:**$O_{2}HB$ signal x(n), Troughs of $O_{2}HB$ signal *K*, and the length of moving average
filter *L*
**Output:**RR1:m(n)← Spline (*K*, x(K), 1 to length(x(n)))
2:MA←$\frac{1}{L}\times$ ones(*L*,1)3:S(n)← filtfilt(MA,1,m(n))
4:G(n)←m(n)−S(n)5:[P,F]← FFT (G(n))
6:r← find(P(F)← Max(P(F)))
7:RR← r × 60
8:**return** 
RR


### 2.3. Evaluation Criteria

The performance of trough and motion-induced artifact detection is assessed using the critical success index (CSI), defined as

(1) $$\mathrm{CSI}=\frac{T_{P}}{T_{P}+F_{N}+F_{P}},$$

where $T_{P}$, $F_{N}$, and $F_{P}$ stand for correctly, missed, and wrongly detected trough and artifactual samples. Regarding RR estimation, the absolute error (AE) between the reference RR obtained from the respiratory signal and estimated RR from $O_{2}Hb$ signal is employed as follows:

(2) $$\mathrm{AE}=|{\mathrm{Reference}}_{RR}-{\mathrm{Estimated}}_{RR}|,$$

To investigate whether there is a significant difference between the estimated and reference RRs, the paired samples t-test with a significance level of 0.05 was performed for each subject.

## 3. Data

In this paper, two different datasets are employed. The first one is used to adjust the parameters of the proposed method, whereas the second one is only used for testing. Two different fNIRS devices were used for each dataset, and the reference respiratory signals were recorded simultaneously using a chest-band with a TMSi SAGA 32+/64+ amplifier (Twente Medical Systems International B.V., Oldenzaal, The Netherlands) at a sampling rate of 4000 Hz.

### 3.1. Data Recording Protocol

**Figure 5:**
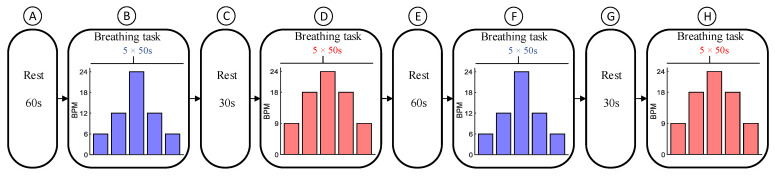
Data recording protocol. It consists of a resting period for 60 s (**A**), and two breathing control tasks lasting for 250 s (**B**,**D**), which are separated by a 30 s resting period (**C**). Subsequently, the same blocks were repeated (**E**–**H**).

Before starting the experiment, the subjects were briefed on the procedure and instructed on how to perform the tasks, in English. The data recording protocol for RR estimation, which was adapted from [32,33,34], is shown in Figure 5. As displayed, it consisted of one block of a resting period lasting for 60 s (A), followed by two blocks of breathing control tasks (B and D), separated by a 30 s rest period (C). Subsequently, the same blocks were repeated (E to H). The subjects were asked to inhale and exhale at a constant pace and at specific rates while watching a bar moving vertically together with a text showing inhale- or exhale phases on the screen. Each block of the breathing control task consisted of 5 steps with a constant RR over a period of 50 s. The RRs for the first and third blocks were 6, 12, 24, 12, and 6 BPM, and for the second and fourth blocks, they were 9, 18, 24, 18, and 9 BPM.

The local ethics committee of Comitatio Bioetico of the University of Pisa approved this study protocol with ref. num. 2/2020. Before starting the experiment, all subjects were informed about the experiment and signed the consent form. All methods were performed based on the guidelines and regulations required by the Declaration of Helsinki. Data were registered at Artinis Medical Systems B.V., Elst, The Netherlands.

### 3.2. fNIRS Systems for Data Collection

#### 3.2.1. Dataset I

**Figure 6:**
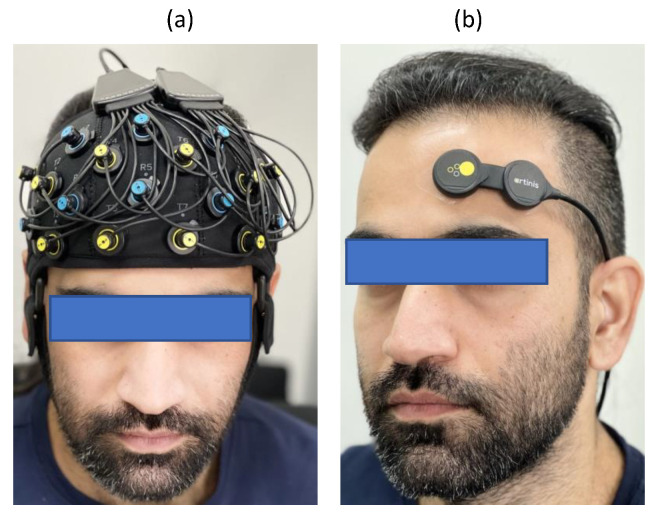
fNIRS optode placement for dataset I (**a**) and dataset II (**b**).

This dataset comprised fNIRS data from 8 healthy subjects (3 female) aged from 21 to 32 years recorded using a portable wireless 23 channel fNIRS system (Brite23, Artinis Medical Systems B.V., The Netherlands) covering the whole frontal cortex (Figure 6a). This device is supplied with a source–detector separation of 35 mm, nominal wavelengths of 760 and 850 nm, ambient light correction, and a sampling frequency of 50 Hz.

#### 3.2.2. Dataset II

This dataset consisted of fNIRS data collected from 18 healthy subjects (9 female) aged from 24 to 37 years using a wireless multi-sensor fNIRS-system (PortaLite MKII, Artinis Medical Systems B.V., The Netherlands). This device is equipped with up to 2 sensors, each having 3 long channels (the source–detector distance being up to 41 mm), and 3 short-separation channels (with distances of 7.2 and 8.0 mm), nominal wavelengths of 760 and 850 nm, ambient light correction, and a sampling rate of 100 Hz. The sensors simultaneously recorded any movement using an IMU embedded within each sensor, and were designed to be placed on both hemispheres of the prefrontal cortex of the brain. In this paper, we have used only a single sensor placed on the left hemisphere of the prefrontal cortex (Figure 6b).

## 4. Experimental Results

In this section, the obtained results from both datasets are described. It is worth mentioning that the required parameters of the proposed method were first tuned based on the optimal results obtained from dataset I, then the adjusted parameters were used for the analysis of dataset II. In addition, the short separation channels of dataset II were discarded to have a similar data structure and analysis for both datasets.

### 4.1. Optimization of the Proposed Method’s Parameters

#### 4.1.1. Trough Detection

**Figure 7:**
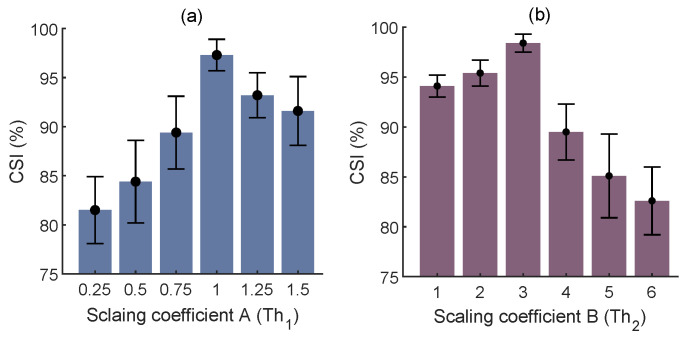
Regulation of constants for trough detection in terms of mean±std of the CSI. $Th_{1}$ (**a**) and $Th_{2}$ (**b**).

Two empirical thresholds require tuning for the trough detection: $Th_{1}$, which is necessary for ignoring the local minima in the dicrotic notch, i.e., a small downward deflection between the peaks and troughs, and $Th_{2}$, which is used to discard the troughs contaminated by the motion artifacts. Regarding the scaling coefficient of $Th_{1}$, values from 0.25 to 1.5 with a step size of 0.25 were inspected. The best fit, i.e., the highest mean CSI, was A=1 (Figure 7a). As for the scaling coefficient of $Th_{2}$, values from 1 to 6 with a step size of 1 were investigated, and B=3 was obtained as the best fit (Figure 7b).

#### 4.1.2. The Length of MA Filtering

As displayed in Figure 4, removing very-low-frequency components of the generated baseline wander is of great importance for the accurate estimation of RR. For this aim, different lengths of the MA filter from 2 to 5 s with a stepping size of 0.5 s were investigated. According to the obtained average AE, although no noticeable difference was observed between different lengths, 3 s had the lowest error (Table 1).

#### 4.1.3. Results of RR Estimation from Dataset I

In total, 160 50 s trials of the concurrent $O_{2}Hb$ and respiratory signals were used, where each RR was repeated 4 times per subject. Table 2 displays the average AE between the reference and estimated RRs for each subject. The average AE of all trials was 2.6 BPM. Given the reported results from the PPG-based studies for RR estimation [32,33,34], the obtained results indicate the feasibility of the proposed method for RR estimation from fNIRS. According to the conducted statistical analysis between the reference and estimated RRs, except for the subject 8, there is no significant difference (p>0.05).

### 4.2. Results of RR Estimation from Dataset II

**Figure 8:**
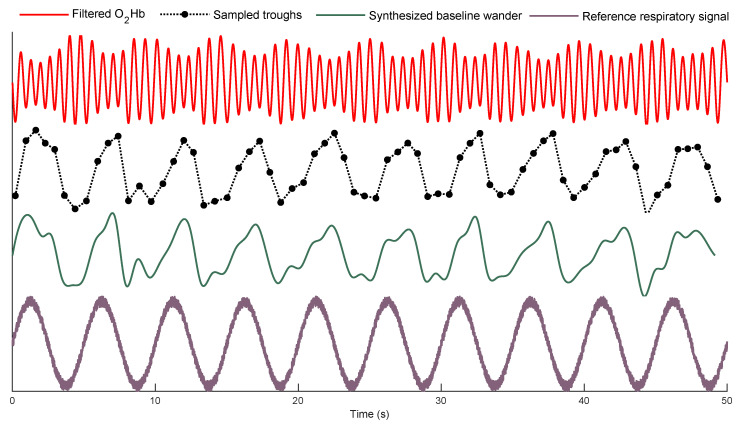
An example of the filtered $O_{2}Hb$ signal, the corresponding baseline wanders, and the reference respiratory signal.

An example of the filtered $O_{2}Hb$ signal, as well as its corresponding extracted baseline wanders and the reference respiratory signal are displayed in Figure 8. As it can be seen, the frequency of baseline wander is close to that of the reference respiratory signal.

**Figure 9:**
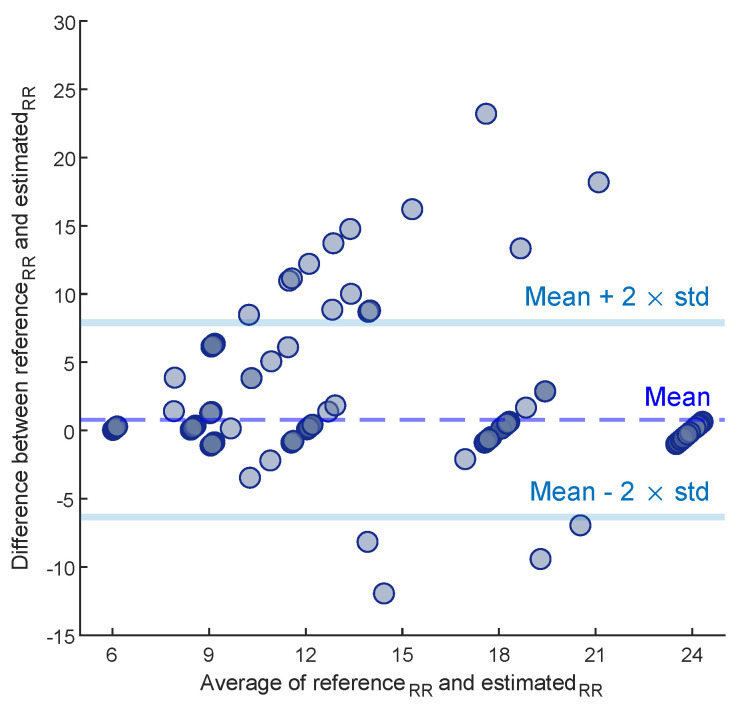
The Bland–Altman plot of the estimated RRs on dataset II.

The average AE between the reference and estimated RRs for each subject of dataset II is disclosed in Table 3. Except for subject 13, the statistical analysis shows no significant difference between the reference and estimated RRs (p>0.05). The average AE for all trials is 1.3 BPM. To evaluate the overall performance of the proposed method on dataset II, a Bland–Altman plot (Figure 9) was used; this assesses the agreement between reference and estimated RRs by showing the difference between each estimate and the references against their mean. In this paper, the Limit of Agreement (LOA) is computed as [mean−2×std,mean+2×std]. In this range, 94% of the differences are inside.

## 5. Discussion

The aim of this paper was to investigate the possibility of RR estimation from fNIRS. Indisputably, this is a novel view on respiration interference in fNIRS, as almost all studies have considered such an interference as a detrimental phenomenon. The importance of this study is to derive an extra measure, i.e., RR, to the cerebral activity analysis without requiring a reference signal. From the research point of view, the proposed method facilitates the approximation of RR in applications where both cerebral and respiratory activities may synergize the analysis. For instance, there is solid evidence in the literature suggesting that RR alternation is an indicator of anxiety and mental workload levels [35,36].

### 5.1. Significance and Robustness of the Proposed Method

It is common knowledge that inhalation and exhalation can alter the blood flow within the body [37]. On the other hand, respiratory fluctuations affect the cerebral blood volume and flow [38]. Therefore, it can be expected that alternation in RR is revealed in fNIRS, and in particular, in $O_{2}Hb$ signals [26]. Our underlying assumption, which was inspired from PPG-based studies [25], was that fluctuations in the frequency of $O_{2}Hb$ signal’s baseline wander can be related to RR.

The proposed method is based on the FFT obtained from the baseline wander of an $O_{2}Hb$ signal. To synthesize the baseline wander, either troughs or peaks can be used. In this paper, we have found the localization of troughs to be more convenient (Figure 2). Yet, synthesizing the baseline wander required an interpolation method due to the irregular sampling. Here, we used cubic spline interpolation, which was already proven as an effective method in PPG-based studies [30]. Afterwards, the dominant frequency of synthesized baseline wander in the FFT domain was considered as RR. The motivation behind using FFT rather than other spectral analyses such as Welch or MUSIC is its simplicity. i.e., the FFT is a non-parametric algorithm.

To assess the feasibility of the proposed method, two different datasets were used: one for adjustment of the required parameters of the proposed method and the other for testing. Indeed, the second dataset was used to investigate the robustness of the proposed method’s parameters when data were recorded from another fNIRS equipment with different characteristics, e.g., the sampling rate, receiver gain, LED types, etc. The comparison between Table 2 and Table 3 confirms such robustness as there is no noticeable difference between the obtained results from both datasets. More surprisingly, even a lower mean of AE was achieved with the second dataset. One plausible explanation can be the weak performance of our method for subject 8 in dataset I.

### 5.2. Comparison with State-of-the-Art Methods

As this is the first research that proposes a method for RR estimation from fNIRS, there is no possibility for comparing the performance of the proposed method to other studies. Yet, a few investigations have aimed to regress out the respiratory components from fNIRS data. For example, Tong et al. [38] used a zero-phase band-pass filter with cut-off frequencies from 0.2 to 0.6 Hz to partition out the respiratory components from the $O_{2}Hb$ signals. In another study, Lühmann et al. [39] proposed a multimodal extension of the general linear model based on temporally embedded canonical correlation analysis to extract respiratory components from fNIRS measurements. The former methodology may not be efficient, as such a band width also involves cerebral activities, and the latter requires the simultaneously recorded respiratory signal. Nonetheless, neither of the mentioned studies have considered RR estimation from the regressed respiratory components. Although it may be inequitable to compare the quality of estimated RR from fNIRS with PPG-based studies, our results are still comparable to [34], where both the finger and forehead PPG data were used.

On the other hand, the mentioned studies used a large amount of data, which is not appropriate for practical applications, as wearing a cap covering the whole head discomforts the user for long-term recording. Furthermore, such a configuration usually involves covering the hair-bearing areas of the head that are more subjected to noise. In contrast, we developed and tested our algorithm-based sensors placed only on the frontal region of head, which provide the user with more comfort, as it is mostly a hairless area. In addition, such a configuration reduces the complexity of wearable instrumentation as only one region of the brain is monitored.

### 5.3. Directions for Future Work

Regardless of the reported promising results, this research has several limitations that should be addressed in future works. Firstly, the employed SQI algorithm is not necessarily an optimal method for finding the best fNIRS data for the analysis. In particular, the SQI is not sensitive to the emergence of motion artifacts, which can significantly influence the synthesis of baseline wander. Yet, to the best of our knowledge, it is one of the best methods for monitoring the quality of data. Secondly, the data recording protocol was a simple breathing task where subjects needed to sit on a chair and perform the experiment. In future work, the subjects should be asked to perform more dynamic activities, e.g., cycling, to obtain more realistic RRs. Thirdly, due to the nature of the data recording protocol, fNIRS data were not significantly contaminated by motion artifacts. Thus, the performance of $Th_{1}$ and $Th_{2}$ for trough detection should be investigated further, with more artifactual data. Fourthly, the performance of the proposed method should be investigated by analyzing different time windows. Fifthly, it should be mentioned that the correction for multiple comparisons has not been conducted for statistical analysis. Lastly, the reliability of the proposed method was only evaluated on young healthy subjects. It is also of great importance to assess the robustness of the proposed method on more diverse cohorts (e.g., the elderly, neonates, and patients) as some studies showed that the performance of RR estimation algorithm can be affected by different factors such as age [40]. Nevertheless, it should be noted that this is the first research that has proposed a method for RR estimation from fNIRS; therefore, having the mentioned limitations was necessary for investigating the possibility of RR estimation.

## 6. Conclusions

In this paper, a method based on the spectrum analysis of the $O_{2}Hb$ signal’s baseline wander was introduced to estimate RR, and its performance was assessed on two different datasets with distinctive data recording characteristics. The comparison between the obtained results from both datasets confirmed the robustness of the proposed method, which is of great importance for real-world applications. The milestone of this research was to consider the respiration interference in fNIRS as source of complementary information, rather than a source of artifact. Indeed, the proposed method can provide extra information from fNIRS that can be used alongside the cerebral activity analysis.

## Figures and Tables

**Table 1:** The Influence of MA Filter’s Length on the RR estimation.

MA Filter Length (s)	Average AE ± Std (BPM)
2	3.2 ± 1.9
2.5	3.1 ± 1.8
3	2.6 ± 1.3
3.5	2.7 ± 1.4
4	2.9 ± 1.9
4.5	2.9 ± 2.1
5	3.1 ± 2.2

**Table 2:** The average AE between the reference and estimated RRs for each subject of dataset I.

Subjects	Average AE (BPM)
1	0.9
2	2.7
3	2.7
4	1.1
5	2.2
6	1.9
7	2.1
8	5.2

**Table 3:** The average of AE between the reference and estimated RRs for each subject of dataset II.

Subjects	Average AE (BPM)
1	1.7
2	0.3
3	0.3
4	0.5
5	1.8
6	0.8
7	1.5
8	2.7
9	2.1
10	0.3
11	0.4
12	0.7
13	3.6
14	0.5
15	1.8
16	1.8
17	0.4
18	2.1

## Data Availability

The data that support the findings of this study are available from the corresponding author, [science@artinis.com], upon reasonable request.
